# HIV risk, risk perception and uptake of HIV testing and counseling among youth men who have sex with men attending a gay sauna

**DOI:** 10.1186/s12981-019-0229-z

**Published:** 2019-06-12

**Authors:** Thana Khawcharoenporn, Suteera Mongkolkaewsub, Chanon Naijitra, Worawoot Khonphiern, Anucha Apisarnthanarak, Nittaya Phanuphak

**Affiliations:** 1 Division of Infectious Diseases, Faculty of Medicine, Thammasat University, Pathumthani, Thailand; 2 Thammasat University Hospital, Pathumthani, Thailand; 3 PREVENTION, The Thai Red Cross AIDS Research Centre, Bangkok, Thailand

**Keywords:** Human immunodeficiency virus, Testing and counseling, Risk perception, Linkage to care, Youth, Men who have sex with men, Thailand

## Abstract

**Background:**

Men who have sex with men (MSM) are amongst populations at-risk for HIV acquisition in Thailand. In youth MSM (aged 15–24 years), the incidence of HIV infection has substantially increased. However, data on HIV risk, risk perception and HIV testing and counseling (HTC) uptake among youth MSM in hotspots are limited.

**Methods:**

A subanalysis of a prospective study among Thai MSM attending a gay sauna was conducted. HIV risk and risk perception were assessed by an anonymous survey. The MSM were categorized as having actual “low-risk”, “moderate-risk” and “high-risk” for HIV acquisition based on the validated study risk categorization tool. HTC was provided on-site with result notification within 1 h. HIV care establishment appointment was arranged by the counselors for HIV-infected participants. Care engagement within 1 year of diagnosis was subsequently assessed.

**Results:**

There were 358 MSM participants; 87 (24%) were youth MSM. Comparing to other MSM, youth MSM had significantly higher median number of lifetime sexual partners [2 (IQR 1–9) vs. 1 (IQR 0–1); *P* < 0.001), were more-likely to ever exchange sex for money (44% vs. 9%; *P* < 0.001) and have sexual partner who exchanged sex for money (8% vs. 1%; *P* < 0.001). Rates of consistent condom use in the past 3 months for anal, oral and vaginal sexes were low and not significantly different between youth and other MSM (51% vs. 61%, 26% vs. 35% and 72% vs. 61%, respectively). By using the study risk categorization tool, there were 68 youth MSM with moderate or high-risk for HIV acquisition, of which 43 (63%) had false perception of low HIV risk. Youth MSM were more likely than other MSM to accept HTC [68% vs. 33%, *P* < 0.001)] and to be first-time testers (42% vs. 28%, *P* = 0.07). By HTC, the rates of HIV infection tended to be higher among youth MSM comparing to other MSM [14/59 (24%) vs. 11/89 (12%); *P* = 0.07]. Among the 14 youth MSM newly-diagnosed with HIV infection, only 6 (43%) showed-up for continuity care after 1-year follow-up.

**Conclusions:**

Youth MSM had substantial high HIV risk, false perception of low HIV risk and low rate of care engagement but demonstrated considerable rate of HTC uptake. Strategies to improve access to HTC, risk perception and linkage to care are needed for HIV prevention and management among the youth MSM.

## Background

Men who have sex with men (MSM) have been significantly affected by HIV infection in Thailand. Despite the overall decline in the prevalence of HIV infection in Thai general population, the HIV prevalence of Thai MSM has increased from 8.0% in 2010 to 9.2% in 2015 [1]. Unawareness of own HIV status and risk behaviors including having multiple sexual partners, high rates of partner change, unprotected sexual intercourse and drug use for sex pleasure were shown to be associated with the ongoing HIV transmission among the MSM population [2]. In the recent observational cohort study [3], young MSM (aged 15–22 years old) were reported to have highest risk for HIV infection among the participating MSM in Bangkok, Thailand with the estimated HIV incidence increasing from 4.1 in 2003 to 7.6 per 100 person-years in 2014. Playing receptive anal sex role and drug use with sex have increased overtime while the rates of consistent condom use and prior HIV test were lower than 50% [3]. These findings suggest the need for more determined and rigorous HIV prevention efforts to stop the HIV epidemic among these young MSM.

The United States Centers for Disease Control and Prevention (CDC) has recommended targeted HIV testing for persons at high-risk for HIV infection including MSM [4]. However, a study from the US revealed that only 28% of young MSM received HIV tests despite that they accounted for 83% of new diagnoses of HIV infection among all youths in non-health care facilities [5]. In addition, another study demonstrated that the rate of HIV-infected young MSM who were unaware of their infection was 52% compared to the rate of 15% among other HIV-infected persons [6]. In Thailand, targeted HIV interventions for MSM including HIV testing and counseling (HTC) and referral for HIV care, condom promotion and distribution, and sexually transmitted infection (STI) treatment, have been implemented nationwide for the past decade. However, the uptake of such interventions and preventive services has been less than 50% since 2010 [1]. The low-level uptake may be partly due to the unmet need for different approaches to reach the MSM, especially those in venue-based settings or those require peer-outreach and social network approaches. These suggest that strategic and innovative approaches to reach and recruit the diverse groups of MSM are required for HIV case finding and prevention. Nonetheless, studies on HIV risk perception and feasibility and uptake of HTC, HIV care referral and preventive services are currently limited among Thai MSM and have not specifically focused on young MSM from hotspots [2, 3].

This study was conducted to assess HIV risks, risk behaviors, HIV transmission prevention knowledge, HIV risk perception, HTC uptake, HIV infection and care engagement among youth MSM in comparison to other MSM from a hotspot.

## Methods

### Study design, population and setting

A subanalysis of the 3-year prospective cohort study among MSM aged at least 18 years old who attended a gay sauna in Thailand was conducted. The original study period was from 1 November 2013 to 31 October 2016 [7]. This study was approved by the Human Ethics Committee of Faculty of Medicine, Thammasat University.

### Study protocol

The original study aims to assess active targeted HTC and linkage to care after known HIV infection among MSM visiting a gay sauna in Thailand [7]. The research team consisted of an Infectious Diseases physician, two HIV counselors, two laboratory technicians, and two volunteers from a gay community-based organization. All MSM attending the gay sauna were approached and asked to participate in the study by the research team upon entering the sauna. Informed consent was obtained from the MSM for both anonymous survey and HTC participation. Study identification numbers derived from the MSM’s initials and year of birth were used to prevent repeat enrollment. Knowledge about HIV infection and transmission prevention was assessed via a survey form which required the MSM to answer “true”, “false” or “do not know” in response to the statements about HIV infection. The knowledge was quantified by the proportion of MSM who responded to each statement correctly. Demographics, HIV risks, risk behaviors and risk perception were collected via a survey form completed by the MSM in a private room.

### HIV testing and counseling procedures

The participating MSM were asked whether they would like to be tested for HIV infection. Reasons for accepting or declining HIV testing were recorded. MSM who accepted HTC were pre-test counseled and asked about their previous HIV status. Known HIV-infected MSM were excluded. Anti-HIV test was performed on-site and all testers were informed of the results within 1 h. Post-test counseling was conducted by the HIV counselors in the team. In order to make follow-up calls for HIV-infected MSM, the primary contact information was recorded. The counselors subsequently discussed with HIV-infected MSM about their plans for HIV continuity care. For follow-up, the counselors called the HIV-infected MSM every month for 1-year period to assess HIV care establishment. Further support and advice for care establishment were provided to those who had not yet established HIV care.

### Study definition

Youth MSM was defined as MSM who were 15–24 years old [8]. Since we included MSM who were 18 years old and older, the youth MSM in this study were 18–24 years old. Sexual orientation and HIV risk perception were self-identified by the MSM within the survey.

If the MSM reported having sex with only male, they were classified as homosexual MSM while those who reported having sex with both sexes were classified as bisexual MSM. The MSM identified their own HIV risks by choosing “low-risk”, “moderate-risk” and “high-risk” in the survey. The investigators then assessed the participants’ risk as “low-risk”, “moderate-risk” and “high-risk” based on the pre-specified risk characteristics and behaviors reported in the survey (Table 1). Only one characteristic or behavior that met the certain risk level was required to classify the participants into that risk level. The participants were classified to have the highest risk level they had. This risk categorization tool was validated among in the previous study for use in differentiating participants with different levels of HIV risks [9]. MSM who had false perception of low HIV risk were those who had moderate or high-risk by the risk categorization tool but perceived their risks as low risk.

**Table 1:** Human immunodeficiency virus (HIV) risk stratification according to the pre-specified reported characteristics and behaviors of the men who have sex with men participants

Characteristics and behaviors	HIV risk		
	Low	Moderate	High
Number of different sexual partners within 30 days			
0–1	√		
2–3		√	
> 3			√
Number of new sexual partners within 30 days			
0–1	√		
2–3		√	
> 3			√
Using condom with vaginal sex			
Always	√		
Most of the time	√		
About a half of time		√	
Sometimes			√
Never			√
Using condom with oral sex			
Always	√		
Most of the time	√		
About a half of time		√	
Sometimes		√	
Never		√	
Using condom with anal sex			
Always	√		
Most of the time	√		
About a half of time		√	
Sometimes			√
Never			√
Exchanging sex for money			
No	√		
Yes			√
Drinking alcohol with sex within 30 days			
Never	√		
Sometimes		√	
About a half of time			√
Most of the time			√
Always			√
Using drug with sex within 30 days			
Never	√		
Sometimes		√	
About a half of time			√
Most of the time			√
Always			√
Ever injected drug with needles			
No	√		
Yes			√
Ever shared needle to inject drugs			
Never	√		
Sometimes			√
About a half of time			√
Most of the time			√
Always			√
Ever been in a jail or a prison			
No	√		
Yes			√
History of STIs within the past year			
No	√		
Yes/not sure			√
Sexual partner had STIs within the past year			
No	√		
Yes/not sure			√
Sexual partner had exchanged sex for money or drugs within 30 days			
No	√		
Yes/not sure			√
Sexual partner had used drug within 30 days			
No	√		
Yes/not sure			√
Sexual partner had been in a jail or a prison			
No	√		
Yes/not sure			√

STIs, sexually transmitted infections

### Data analyses

Characteristics, HIV risks and risk behaviors, knowledge about HIV infection and transmission prevention, HIV risk perception, HTC acceptance and linkage to care were compared between youth and other MSM. All statistical analyses were performed using SPSS version 15.0 (SPSS, Chicago, Illinois). Categorical variables were compared using Pearson’s χ^2^ or Fisher’s exact test as appropriate. Continuous variables were compared using Mann–Whitney U test. All *P* values were 2 tailed; *P* values less than 0.05 were considered statistically significant. Variables associated with declining HTC and HIV infection with a significance level of P < 0.20 were entered into multivariable logistic regression model in stepwise backward fashion. Significant variables that were thought to be covariates were grouped, and only one variable from each group was chosen for model entry. The model’s overall robustness was confirmed by Hosmer–Lemeshow goodness-of-fit statistic. Adjusted odd ratios (aORs) and 95% confidence intervals (CIs) were calculated for risk factors associated with declining HTC and HIV infection.

## Results

### Characteristics and HIV knowledge of the study participants

A total of 358 MSM participated in the original study. Demographics characteristics of the participating MSM are shown in Table 2. Most of the MSM were company workers, single, and originally from Bangkok, had highest education of bachelor degree or higher and had monthly household income of $US 1800 or less. Of the 358 MSM, 87 (24%) were youth MSM. Comparing between youth and other MSM, youth MSM were more likely to be college or university students, originally from outside Bangkok, had lower highest education level and monthly household income (Table 2). In regards to knowledge about HIV, most of the MSM (≥ 80%) responded to the survey statements correctly (Table 3), except for the statements “you can get HIV from oral sex” and “a vaccine that can prevent HIV is currently available”. Significantly less proportion of the youth MSM compared to other MSM correctly responded to the statements “a mosquito can transmit HIV”, “you can get HIV from dining with an infected person”, “getting high by using drugs increases risk of getting HIV”, “you can get HIV from tattooing” and “a vaccine that can prevent HIV is currently available” (Table 3).

**Table 2:** Demographic characteristic of men who have sex with men (MSM) participants

Characteristic	All (N = 358)	Youth MSM (N = 87)	Other MSM (N = 271)	*P* ^a^
Occupation				*<* *0.001*
Company worker	203 (57)	35 (40)	168 (62)	
Merchant	54 (15)	1 (1)	53 (20)	
College/University student	51 (14)	42 (48)	9 (3)	
Government officer	39 (11)	4 (5)	35 (13)	
Unemployed	10 (2.7)	5 (6)	5 (1.7)	
Housemaid	1 (0.3)	0 (0)	1 (0.3)	
Birthplace				*0.03*
Bangkok	169 (47)	31 (36)	138 (51)	
Central Western and Eastern Thailand	73 (20)	16 (19)	57 (21)	
Northeastern Thailand	60 (17)	22 (25)	38 (14)	
Northern Thailand	42 (12)	28 (16)	28 (10)	
Southern Thailand	14 (4)	10 (5)	10 (4)	
Marital status				0.78
Single	264 (74)	67 (77)	197 (73)	
Living separate with partner	49 (13)	11 (13)	38 (14)	
Living with domestic partner	31 (9)	7 (8)	24 (9)	
Married	14 (4)	2 (2)	12 (4)	
Highest education				*<* *0.001*
Less than primary school	7 (2)	5 (6)	2 (0.7)	
Primary school	8 (2)	7 (8)	1 (0.3)	
High school	101 (28)	40 (46)	61 (23)	
Bachelor’s degree	177 (50)	33 (38)	144 (53)	
Higher than bachelor’s degree	65 (18)	2 (2)	63 (23)	
Monthly household income				*<* *0.001*
Less than $US450	37 (10)	23 (26)	14 (5)	
$US450–$US1800	182 (51)	48 (55)	134 (49)	
$US1801–$US4500	84 (24)	6 (7)	78 (29)	
More than $US4500	55 (15)	10 (12)	45 (17)	

Data are in numbers (%) unless otherwise indicated

^a^Comparison between youth MSM and other MSM

**Table 3:** Knowledge about HIV infection among the men who have sex with men (MSM) participants

Statement (correct answer)	All (N = 358)	Youth MSM (N = 87)	Other MSM (N = 271)	*P* ^a^
HIV infection causes by a virus (True)	350 (98)	84 (97)	266 (98)	0.41
A mosquito can transmit HIV (False)	286 (80)	60 (69)	226 (83)	*0.003*
You can get HIV from dining with an infected person (False)	309 (86)	67 (77)	242 (89)	*0.004*
You can get HIV from vaginal sex (True)	336 (94)	84 (97)	252 (93)	0.31
You can get HIV from anal sex (True)	343 (96)	83 (95)	260 (96)	0.77
You can get HIV from oral sex (True)	275 (77)	65 (75)	210 (78)	0.59
Having multiple sexual partners increases risk of getting HIV (True)	348 (87)	84 (97)	264 (97)	0.71
Consistent condom use with sex decreases risk of getting HIV (True)	353 (99)	86 (99)	267 (99)	1.00
Exchanging sex for money increases risk of getting HIV (True)	340 (95)	82 (94)	258 (95)	0.72
Getting high by using drugs increases risk of getting HIV (True)	294 (82)	64 (74)	230 (85)	*0.02*
You can get HIV from tattooing (True)	320 (90)	72 (83)	249 (92)	*0.02*
You can get HIV from using a shared needle (True)	349 (98)	85 (98)	264 (97)	1.00
An HIV-infected person can be asymptomatic for many years (True)	327 (91)	79 (91)	248 (92)	0.84
An asymptomatic HIV-infected person can transmit HIV (True)	336 (94)	84 (97)	252 (93)	0.31
A blood test is required for HIV diagnosis (True)	292 (82)	68 (78)	224 (83)	0.35
A vaccine that can prevent HIV is currently available (False)	151 (42)	25 (29)	126 (37)	*0.004*
Antiretroviral therapy can increase lifespan of an HIV-infected person (True)	304 (85)	71 (82)	233 (86)	0.32

Data are in numbers (%) of participants with a correct answer for each statement

^a^Comparison between youth and other MSM

### HIV risks, risk behaviors and risk perception of the study participants (Table 4)

Of the 358 MSM, 58% were homosexual and 17% reported history of exchanging sex for money. The rates of consistent condom use for vaginal, oral and anal sex were 64%, 33% and 59%, respectively. Among the 151 MSM who reported drinking alcohol within 30 days, 75 (50%) reported drinking alcohol with sex. Twenty-two MSM reported having STIs within the past year, of which 12 (55%) had gonorrhea. Compared to other MSM, youth MSM had significantly higher median number of new and different sexual partner within the last month (2 vs. 1; P < 0.001), were more-likely to exchange sex for money (44% vs. 9%; P < 0.001) and have sexual partner who exchanged sex for money (8% vs. 1%; P < 0.001). By using the study risk categorization tool, 262 of the 358 MSM had moderate or high risk for HIV acquisition, of which 172 (66%) had false perception of low HIV risk. The rates of false perception of low HIV risk were not significantly different between youth MSM and other MSM (63% vs. 66%). Among MSM with low HIV risk, only 1 of 19 (5%) youth MSM and none of other MSM perceived themselves at high-risk for HIV acquisition.

**Table 4:** HIV risk, risk behaviors and risk perception of all men who have sex with men (MSM) participants

Characteristics	All (N = 358)	Youth MSM (N = 87)	Other MSM (N = 271)	*P* ^a^
Sexual orientation				0.50
Homosexual	207 (58)	53 (61)	154 (57)	
Bisexual	151 (42)	34 (39)	117 (43)	
Number of different sexual partners for the last 1 month (median, IQR)	1 (1–3)	2 (1–9)	1 (0–2)	*<* *0.001*
Number of new sexual partners for the last 1 month (median, IQR)	1 (1–2)	2 (1–9)	1 (0–2)	*<* *0.001*
Having vaginal sex	135 (38)	32 (37)	103 (38)	0.84
Condom use with vaginal sex				*0.04*
Always	86/135 (64)	23/32 (72)	63/103 (61)	
Most of the time	25/135 (19)	3/32 (9)	22/103 (21)	
About a half of time	9/135 (7)	5/32 (16)	4/103 (4)	
Sometimes	8/135 (6)	1/32 (3)	7/103 (7)	
Never	7/135 (5)	0/32 (0)	7/103 (7)	
Having oral sex	316 (88)	77 (89)	239 (88)	0.94
Condom use with oral sex				0.10
Always	103/316 (33)	20/77 (26)	83/239 (35)	
Most of the time	66/316 (21)	20/77 (26)	46/239 (19)	
About a half of time	40/316 (13)	13/77 (17)	27/239 (11)	
Sometimes	62/316 (20)	18/77 (23)	44/239 (18)	
Never	45/316 (14)	6/77 (8)	39/239 (16)	
Having anal sex	345 (96)	87 (100)	258 (95)	*0.04*
Condom use with anal sex				0.32
Always	202/345 (59)	44/87 (51)	158/258 (61)	
Most of the time	68/345 (20)	18/87 (21)	50/258 (20)	
About a half of time	37/345 (11)	14/87 (16)	23/258 (9)	
Sometimes	28/345 (8)	8/87 (9)	20/258 (8)	
Never	10/345 (3)	3/87 (3)	7/258 (3)	
Exchanging sex for money	62 (17)	38 (44)	24 (9)	*<* *0.001*
Drinking alcohol with sex within 30 days				0.40
Never	76/151 (50)	26/46 (57)	50/105 (48)	
Sometimes	42/151 (28)	12/46 (26)	30/105 (29)	
About a half of time	17/151 (11)	5/46 (11)	12/105 (11)	
Most of the time	12/151 (8)	1/46 (2)	11/105 (11)	
Always	4/151 (3)	2/46 (4)	2/105 (4)	
Using drug with sex within 30 days				0.61
Never	3/13 (23)	1/4 (25)	2/9 (22)	
Sometimes	7/13 (54)	3/4 (75)	4/9 (44)	
About a half of time	2/13 (15)	0/4 (0)	2/9 (22)	
Most of the time	0/13 (0)	0/4 (0)	0/9 (0)	
Always	1/13 (8)	0/4 (0)	1/9 (11)	
History of STIs within the past year				0.94
Yes	22 (6)	6 (7)	16 (6)	
Not sure	13 (4)	3 (3)	10 (4)	
No	323 (90)	78 (90)	245 (90)	
Type of STIs within the past year^b^				
Gonorrhea	12/22 (55)	4/6 (67)	8/16 (50)	0.65
Herpes simplex infection	6/22 (27)	2/6 (33)	4/16 (25)	1.00
Genital wart	4/22 (18)	0/6 (0)	4/16 (25)	0.54
Syphilis	1/22 (5)	0/6 (0)	1/16 (7)	1.00
Unknown	1/22 (5)	0/6 (0)	1/16 (7)	1.00
Sexual partner had STIs within the past year				0.86
Yes	18 (5)	4 (5)	14 (5)	
Not sure	107 (30)	28 (36)	79 (29)	
No	233 (65)	55 (63)	178 (66)	
Type of STIs within the past year of sexual partner^b^				
Gonorrhea	8/18 (44)	3/4 (75)	5/14 (36)	0.25
HIV	6/18 (33)	0/4 (0)	6/14 (43)	0.25
Genital wart	2/18 (11)	0/4 (0)	2/14 (14)	1.00
Herpes simplex infection	1/18 (6)	1/4 (25)	0/14 (0)	0.22
Syphilis	1/18 (6)	0/4 (0)	1/14 (7)	1.00
Unknown	8/18 (44)	3/4 (75)	5/14 (36)	1.00
Sexual partner had exchanged sex for money within 30 days				*<* *0.001*
Yes	9 (3)	7 (8)	2 (1)	
Not sure	89 (25)	24 (28)	65 (24)	
No	260 (72)	56 (64)	204 (75)	
Sexual partner had used drug within 30 days				0.31
Yes	6 (2)	3 (2)	3 (1)	
Not sure	87 (24)	51 (35)	36 (17)	
No	265 (74)	62 (71)	203 (75)	
Perceiving own HIV risk as				0.33
No or low risk	267 (74)	61 (70)	206 (76)	
Moderate risk	73 (20)	22 (25)	51 (19)	
High risk	18 (5)	4 (5)	14 (5)	
HIV risk determined by the study tool				0.14
Low risk	96 (27)	19 (22)	77 (28)	
Moderate risk	56 (16)	10 (12)	46 (17)	
High risk	206 (58)	58 (67)	148 (55)	
False perception of low HIV risk	172/262 (66)	43/68 (63)	129/194 (66)	0.63
Correct risk perception among MSM with low HIV risk	95/96 (99)	18/19 (95)	77/77 (100)	0.20

Data are in numbers (%) unless otherwise indicated

HIV, human immunodeficiency virus; IQR, interquartile range; STI, sexually-transmitted infection

^a^Comparison between youth and other MSM

^b^One participant could have more than one type of STIs

### HIV testing and counseling acceptance

Of the 358 MSM in this study, 210 declined HTC. Significantly less proportion of youth MSM than other MSM declined HTC (32% vs. 67%; P < 0.001). The three most common reasons for declining HTC for youth MSM were prior HIV test within 6 months (50%), not ready (36%) and considering the gay sauna as an inappropriate place for HIV testing (7%), while the three most common reasons for other MSM were prior HIV test within 6 months (47%), not ready (16%) and perceiving no risk for HIV infection (13%) (Table 5). Other reasons for declining HTC are shown in Table 5. When excluding MSM who reported having prior HIV test within 6 months, youth MSM were more likely than other MSM to accept HTC (68% vs. 33%) and to be first-time testers (42% vs. 28%). In multivariable logistic regression analysis adjusted by education level, birthplace and monthly household income, self-perceived low HIV risk (aOR 2.18; 95% CI 1.11–4.29: *P* = 0.02) and low HIV risk as defined by the study risk categorization tool (aOR 2.33; 95% CI 1.17–4.62: *P* = 0.02) increased the likelihood of MSM to decline HTC, while being youth reduced that chance (aOR 0.31; 95% CI 0.15–0.62: *P* = 0.01) (Table 6). Other characteristics including occupation, marital status and sexual orientation were not associated with declining HTC.

**Table 5:** Reasons for declining HIV testing and counseling among the men who have sex with men (MSM) participants

Reason	All (N = 210)	Youth MSM (N = 28)	Other MSM (N = 182)	*P* ^a^
Prior HIV test within 6 months	100 (48)	14 (50)	86 (47)	0.79
Not ready	40 (19)	10 (36)	30 (16)	*0.002*
Perceiving no risk for HIV infection	23 (11)	0 (0)	23 (13)	*0.05*
Inappropriate testing place	18 (9)	2 (7)	16 (9)	1.00
Time constraints	15 (7)	1 (4)	14 (8)	0.70
Being afraid to know test result	8 (4)	1 (4)	7 (4)	1.00
Being afraid of needle	5 (2)	0 (0)	5 (3)	1.00
Being healthy	1 (0.5)	0 (0)	1 (0.5)	1.00

Data are in numbers (%)

^a^Comparing between youth and other MSM

**Table 6:** Factors associated with declining HIV testing and counseling (excluding participants reporting prior HIV test within 6 months)

Risk factors	Univariable analysis		Multivariable analysis	
	OR (95% CI)	*P*	aOR (95% CI)	*P*
Youth MSM	0.23 (0.12–0.43)	*<* *0.001*	0.31 (0.15–0.62)	*0.01*
Perceiving own HIV risk as low risk	3.14 (1.70–5.82)	*<* *0.001*	2.18 (1.11–4.29)	*0.02*
Low HIV risk by the study tool	3.24 (1.75–6.00)	*<* *0.001*	2.33 (1.17–4.62)	*0.02*
Having highest education of bachelor degree and higher	2.35 (1.39–3.98)	*0.001*	1.35 (0.74–2.46)	0.34
Being from Bangkok	2.14 (1.30–3.54)	*0.003*	1.72 (0.99–2.99)	0.06
Monthly household income more than $US 4500	2.37 (1.10–5.11)	*0.03*	1.67 (0.71–3.92)	0.24

aOR, adjusted odds ratio; CI, confidence interval; HIV, human immunodeficiency virus; MSM, men who have sex with men; OR, odds ratio

### HIV test outcomes and linkage to care

Of the 148 MSM accepting HTC, 50 were first-time testers and 25 (17%) had HIV infection (Table 7). There was a trend toward significance of higher proportion of first-time testers and HIV-infected persons among youth MSM compared to other MSM [(42% vs. 28%; *P* = 0.07) and (24% vs. 12%; *P* = 0.07, Table 7). In multivariable logistic regression analysis adjusted by HIV risk determined by the study tool, education level and MSM group, factors associated with HIV infection were false perception of low HIV risk (aOR 3.81; 95% CI 1.37–10.62: *P* = 0.01) and monthly household income of less than $US 450 (aOR 3.16; 95% CI 1.03–9.67: *P* = 0.04) (Table 8). Of the 25 MSM who were newly diagnosed with HIV infection, 12 (48%) established HIV continuity care within 1 year of the diagnosis at a median (interquartile range) time from diagnosis to HIV care of 24 (6–71) days. There was no significant difference between youth MSM and other MSM in proportion of persons who established HIV continuity care (Table 7). Given the small number of MSM who could be assessed for linkage to care (N = 25), we did not analyze and determine factors association with linkage to care.

**Table 7:** HIV testing and counseling, test results and linkage to care among the men who have sex with men (MSM) participants

Characteristic	All (N = 358)	Youth MSM (N = 87)	Other MSM (N = 271)	*P* ^a^
HIV voluntary testing and counseling accepting participants	148 (41)	59 (68)	89 (33)	*<* *0.001*
First-time tester	50/148 (34)	25/59 (42)	25/89 (28)	0.07
Repeat tester	98/148 (66)	34/59 (58)	67/89 (72)	
HIV test result				0.07
Reactive	25/148 (17)	14/59 (24)	11/89 (12)	
Non-reactive	123/148 (83)	45/59 (76)	78/89 (88)	
Linkage to HIV care				0.65
Yes	12/25 (48)	6/14 (43)	6/11 (55)	
No	13/25 (52)	8/14 (57)	5/11 (45)	

Data are in numbers (%)

^a^Comparison between youth MSM and other MSM

**Table 8:** Factors associated with having HIV infection among 148 participants undergoing HIV testing and counseling

Risk factors	Univariable analysis		Multivariable analysis	
	OR (95% CI)	*P*	aOR (95% CI)	*P*
False perception of low HIV risk	4.05 (1.51–10.83)	*0.005*	3.81 (1.37–10.62)	*0.01*
Monthly household income less than $US 450	3.51 (1.34–9.20)	*0.01*	3.16 (1.03–9.67)	*0.04*
Having highest education less than bachelor degree	2.63 (1.11–6.54)	*0.04*	1.63 (0.57–4.66)	0.37
High HIV risk by the study tool	3.16 (0.89–11.19)	0.07	1.90 (0.50–7.20)	0.08
Youth MSM	2.21 (0.92–5.27)	0.08	2.26 (0.90–5.66)	0.34

aOR, adjusted odds ratio; CI, confidence interval; HIV, human immunodeficiency virus; MSM, men who have sex with men; OR, odds ratio

## Discussion

The study findings indicated that significantly higher proportion of youth MSM than other MSM accepted HTC during our outreach program to promote HIV “Test and Treat” and HIV prevention at the gay sauna and being youth MSM was identified as an independent factor associated with HTC acceptance in our multivariable analysis. In addition, a significant number of these youth MSM were first-time testers. These findings suggest the feasibility and opportunity to scale up HTC, linkage to care and HIV transmission prevention programs among at-risk youth MSM in hotspot settings in Thailand. Being unready to be tested was the main reason among MSM who declined and had not had HTC in the past 6 months. This lack of readiness could be related to issues regarding the need for more time to prepare themselves in order to know and accept their HIV test results. This reason was reported more among youth MSM. Thus, strategies such as spending more time in activities to prepare and educate youth MSM for HTC during the outreach program may be necessary. Other factors previously reported to be associated with HTC declining among young MSM were non-employment status, high HIV stigma score, low education level and low knowledge about HIV transmission prevention [10–12] while barriers to HTC included lack of awareness or knowledge about HIV testing, fear of result, fear of rejection, fear of disclosure, limited access to HTC, and unfriendly environment of HIV testing places [13, 14]. Altogether, the associated factors and reasons for HTC declining as well as barriers to HTC need to be considered for implementing HTC and transmission prevention program among youth MSM. Our HTC outreach program could serve as a model to bring out the awareness of and access to friendly HTC services to MSM.

Our study revealed that most of the youth MSM correctly responded to the survey statements indicating general high level of knowledge about HIV infection. However, there were specific topics (proportion of correct responses to the statements) that youth MSM had less knowledge than other MSM. These included routes of HIV transmission (69–77%), drug use and risk of HIV acquisition (74%) and HIV vaccine (29%). Education emphasized on these specific topics should be provided for youth MSM during the outreach program. Despite the high level of knowledge of HIV infection, several HIV risks and risk behaviors among youth MSM were reported in this study. The rates of consistent condom use for oral and anal sex were about 50% or less and were not significantly different between youth and other MSM. Nonetheless, youth MSM had significantly more different and new sexual partners within the past month and higher proportion of them exchanged sex for money and had sexual partner who exchanged sex for money compared to other MSM. The low rate of consistent condom use was comparable to the rate of 47% reported among MSM samples in big cities of Thailand [15] while the high number of sexual partner and the high rate of exchanging sex for money were consistent with the results from a Thai study among MSM recruited from gay entertainment sites and community-based organizations [11]. These findings suggest the need for interventions to reduce HIV risk behaviors among youth MSM and to improve application of their existing knowledge to reduce risk behaviors. Potential effective interventions may include culturally-relevant role model stories, peer outreach, and highly-interactive group-level behavioral interventions tailored to youth MSM [16–18].

In this study, the rate of false perception of low HIV risk among moderate and high-risk youth MSM was high (63%) and was comparable to that of other MSM. The high rates of false perception of low HIV risk were also reported among youth MSM form the US studies [19, 20].

In addition, we identified false perception of low HIV risk as an independent factor associated with HTC declining and HIV infection among youth MSM. These findings underlie the need for HIV risk perception assessment during the outreach program and the importance of accurate perception of HIV risk in facilitating interventions to prevent HIV transmission.

The overall rate of HIV infection among MSM in this study was 17% which was higher than the prevalence of 9% in general Thai MSM population [1]. The higher rate of infection represents the higher risk for HIV acquisition among MSM attending the gay sauna. It should be noted that youth MSM had a higher rate of HIV infection than other MSM (24% vs. 12%). This result was consistent with previous studies that reported young MSM (aged 18–30 years) to be significantly associated with HIV infection [3, 21, 22]. The high prevalence of HIV among youth MSM could be due to higher HIV risks and risk behaviors among this population and lower level of knowledge about HIV transmission prevention and how to prevent self from getting HIV. The other factor associated with HIV infection was low monthly household income, consistent with another Thai study conducting among MSM who came for HIV testing at the Anonymous Clinic and two drop-in centers in Bangkok [22]. The finding suggests youth MSM with lower socioeconomic status to be prioritized for innovative approaches to promote awareness and uptake of HTC.

Despite the on-site face-to-face post-test counseling and advice on plan for long-term HIV care, and the subsequent follow-up calls, less than half of the HIV-infected MSM were established continuity care within 1 year of diagnosis, especially youth MSM. This finding was in accordance with another study that reported young MSM took longer time to entry HIV care after the diagnosis than other population groups [23]. Thus, reasons for no care establishment and barriers to linkage to care require further studies among these youth MSM. Effective strategies to improve linkage to HIV continuity care may include remind messaging via mobile phone and social media, case management, use of incentives for linkage to care, on-site point-of-care CD4 testing, on-site or same-day antiretroviral therapy and education with more emphasis on the importance of linkage to care, antiretroviral therapy adherence and care retention [24–27].

This is a single-site study aiming to represent a suburban hotspot setting in Thailand. The study results may not be generalizable to youth MSM who attend other saunas in Thailand. However, the findings are of importance and address critical issues on HIV risks and risk behaviors, HIV risk perception, HTC acceptance rate of HIV infection and barriers to HTC implementation and linkage to care. These issues need to be focused while implementing outreach programs for HIV prevention among youth MSM attending gay hotspots in other settings.

## Conclusions

Youth MSM were at-higher risk of HIV infection compared to other MSM due to their significant risk behaviors and false perception of low HIV risk. However, the higher rate of HTC uptake among youth MSM should provide a great opportunity to implement and scale up HTC and HIV transmission prevention programs among this population. The HIV transmission prevention programs should incorporate HIV risk perception assessment and interventions to correct the risk perception among the youth MSM. Further studies are needed to assess interventions to improve HTC acceptance, HIV and linkage to HIV care and to determine strategies to implement successful HIV transmission prevention programs among youth MSM attending gay hotspots in Thailand.

## Data Availability

All data generated or analysed during this study are included in this published article and its additional files.

## Abbreviations

AIDS: acquired immune deficiency syndrome
HIV: human immunodeficiency virus
HTC: HIV testing and counseling
MSM: men who have sex with men
STI: sexually-transmitted infection
